# Changes in Hospitalization for Eating Disorders and Related Suicidal Risk, Following COVID‐19 Pandemic

**DOI:** 10.1155/da/3364565

**Published:** 2026-03-03

**Authors:** J. C. Chauvet-Gélinier, E. Lajeune, J. Cottenet, J. M. Pinoit, F. Jollant, C. Quantin

**Affiliations:** ^1^ INSERM Unit, U-1231, Center for Translational and Molecular Medicine, Université Bourgogne Europe, Dijon, France, u-bourgogne.fr; ^2^ Department of Psychiatry, Dijon University Hospital (CHU), Dijon, France; ^3^ Service de Biostatistiques et d’Information Médicale (DIM), CHU Dijon Bourgogne, Dijon, France, chu-dijon.fr; ^4^ Faculty of Medicine, Université Paris-Saclay, Le Kremlin-Bicêtre, France, universite-paris-saclay.fr; ^5^ Service de Psychiatrie, APHP, Hôpital Paul-Brousse, Villejuif, France, aphp.fr; ^6^ McGill Group for Suicide Studies, Department of Psychiatry, McGill University, Montréal, Quebec, Canada, mcgill.ca; ^7^ UVSQ, Inserm, CESP, Université Paris-Saclay, Villejuif, 94807, France, universite-paris-saclay.fr

**Keywords:** anorexia nervosa, atypical eating disorders, binge eating disorder, bulimia nervosa, COVID-19, eating disorders, SARS-CoV-2, self-harm, suicidal behavior

## Abstract

**Objective:**

This study aimed (1) to assess changes in the frequency and severity of eating disorders (EDs) during the COVID‐19 pandemic (March 2020–December 2024) compared to the pre‐pandemic period (January 2015–February 2020) and (2) to evaluate changes in rates of self‐harm and suicidal ideation among individuals with EDs.

**Method:**

Data were extracted from the French National Hospital Discharge Database (PMSI), including all patients hospitalized with a diagnosis of ED between 2015 and 2024 (*N* = 162,621). Negative binomial regression models were used, stratified by age group, to examine the impact of the pandemic on ED hospitalizations. The severity of EDs during the initial stay was studied based on admission to the intensive care unit or the presence of medical complications. The proportion of patients with self‐harm and/or suicidal ideation within 2 years following an ED‐related hospitalization was also estimated, and the changes among the periods were analyzed.

**Results:**

Compared to the pre‐pandemic period, there was a significant increase in ED hospitalizations during the pandemic period, particularly among adolescents and young adults (ages 0–14, 15–19, and 20–24). The proportion of patients hospitalized for self‐harm and/or suicidal ideation within 2 years of an ED stay was higher during the pandemic period (13.31% vs. 8.67%, *p*  < 0.01). The difference in proportion based on severity criteria is significant, with a higher percentage for the post‐COVID period.

**Discussion:**

This nationwide study reveals a marked rise in both the incidence and severity of EDs during the COVID‐19 pandemic, despite the limitations associated with the use of an administrative database. These findings highlight the urgent need for age‐targeted prevention strategies and early intervention programs, particularly for younger populations.

## 1. Introduction

The COVID‐19 pandemic has had a profound impact on mental health worldwide, leading to a surge in psychological distress, including anxiety, depression [1, 2], and even self‐harming behaviors [3, 4], particularly among younger populations [5, 6]. Five years later, there is still a need to better understand the factors contributing to the worsening of psychiatric health in this context. This need is particularly relevant considering that new pandemics are expected to emerge in the future.

Among the various disorders exacerbated by the pandemic, eating disorders (EDs) have drawn significant attention due to their increasing prevalence and severity [7]. Research indicates that lockdown measures, social isolation, and uncertainty contributed to a rise in ED cases, with hospital‐based care reflecting this trend. In France, a sharp increase in hospitalizations for anorexia nervosa (AN) was observed after March 2020, peaking in March 2021, with rates increasing by nearly 50% among adolescent or very young adult populations compared to pre‐pandemic levels [8]. Studies further suggest that, beyond exacerbating pre‐existing EDs, the pandemic also contributed to the emergence of new cases, particularly among high‐risk populations such as adolescents and young adults [9]. One of the most striking findings is the substantial increase in hospital admissions for EDs during the pandemic [10]. In addition to the well‐documented metabolic complications of EDs [11], the issue of suicidal behaviors remains particularly concerning [7, 12]. Suicide is a leading cause of death among individuals with AN [13], while suicidal behaviors are frequently observed in bulimia nervosa (BN) and binge eating disorder (BED) [14]. Studies indicate that between one‐quarter and one‐third of individuals with AN, BN, or BED have experienced suicidal thoughts, and a similar proportion of those with AN and BN have attempted suicide [14]. These findings underscore the critical need for a deeper understanding of the complex interplay between EDs and suicide risk, particularly in the wake of the COVID‐19 pandemic.

In the current study, our aims were twofold: i) investigate changes in the frequency of EDs over the first years of the COVID‐19 pandemic (March 2020–December 2024) as compared to the pre‐pandemic period (January 2015–February 2020) in terms of patients with an identified ED, and ii) explore changes in the rates of self‐harm and suicidal ideation in people with an ED, as well as the severity of ED. We hypothesized that rates of ED increased between 2015 and 2024, as previously reported, but also that rates of patients who self‐harmed increased, suggesting increasing rates of more severe cases of ED. Our research was based on an analysis of France’s exhaustive medico‐economic health databases, covering a 10‐year period from 2015 to 2024.

## 2. Materials and Methods

### 2.1. Database

This study was based on the French national hospital database (Programme de Médicalisation des Systèmes d’Information, PMSI), which was designed to include discharge abstracts for all inpatient admissions to public and private hospitals in France. Inspired by the American diagnosis‐related group (DRG) model, the information in the PMSI abstracts is pseudonymized. Diagnoses identified during the hospital stay are coded according to the 10th edition of the International Classification of Diseases (ICD‐10). Medical procedures performed during hospitalization are coded according to the Common Classification of Medical Procedures (CCAM). The use of this database for the allocation of hospital budgets encourages improvement in data quality in terms of coherence, accuracy, and exhaustiveness [15, 16], in particular in the field of EDs [17] and suicidal risk [18, 19].

### 2.2. Population and Main Events

We included all patients hospitalized between 2015 and 2024 for ED identified with primary, related, or associated diagnosis ICD‐10 codes. We separated ED into different categories: AN, BN, atypical EDs, and BED. The severity of ED is identified during the ED stay by admission to the intensive care unit (ICU), length of stay, and medical complications as a composite event including malnutrition, hypokalemia, liver failure, heart failure, hypoglycemia, kidney failure, leukopenia, neutropenia, hypothermia, change in blood pressure, and placement of a gastric tube. The severity of ED was also identified by self‐harm and/or suicidal ideation in the 2 years following an ED stay (including the ED stay). The ICD‐10 codes and CCAM codes used are detailed in Table S1.

### 2.3. Covariates

Covariates included the year of admission (2015 to 2024) and patient age and sex. The years of admission were divided into three periods for the descriptive analysis (January 2015–February 2020 (pre‐pandemic period), March 2020–May 2023 (pandemic period), and June 2023–December 2024 (early post‐pandemic period)), but only into two periods for the multivariate modeling due to insufficient sample size for the latest period. Age was divided into several categories (0–14, 15−19, 20–24, 25−44, 45–64, and 65 and over), which are regularly used in this field. We also considered depression, psychiatric disorders, and the Charlson score.

### 2.4. Statistical Analyses

We first estimated the rate of change in the number of patients between 2015 and 2024 with an identified ED. We then plotted changes in the number of patients during the period (2015–2024) for each event (ED and their different categories), separating by sex and age groups. A trend line has been added to each graph. This trend was calculated between 2015 and 2019 and used to compare the expected pandemic trend with the observed pandemic trend in order to identify the populations negatively impacted by the COVID‐19 pandemic period.

To determine the impact of the pandemic period (2020–2024) on the number of patients for each event, we performed negative binomial regression, estimating adjusted rate ratios (aRRs). These models were used for each age group, adjusting for sex, depression, psychiatric disorders, and the Charlson score. We also added a social deprivation index [20] as an adjustment factor.

We also compared the severity of EDs (admission to ICU, length of stay, and medical complications) during the three periods studied (pre‐pandemic, pandemic, and early post‐pandemic).

Finally, we compared the rate of suicidal ideation and/or self‐harm in the 2 years following an ED stay, including the ED stay, between the pre‐COVID‐19 period (2015–2017) and the COVID‐19 period (March 2020–December 2022). The periods were selected to ensure a 2‐year follow‐up period outside the COVID‐19 pandemic period for the pre‐COVID‐19 period (2015–2017) and included in the pandemic period for the COVID‐19 period (March 2020–December 2022).

The statistical significance threshold was set at < 0.05. All analyses were performed using SAS (SAS Institute Inc., Version 9.4, Cary, NC).

## 3. Results

### 3.1. Temporal Trends in the Number of Patients With an ED

This study included 162,621 patients hospitalized for ED in France between 2015 and 2024 (Figure S1). For each event, the number of patients increased over the study period: +56.38% for ED overall, +42.73% for AN, +26.6% for BN, +80.16% for atypical ED, and +158.32% for BED.

Analysis of ED patient characteristics (overall or by category) (Table 1) highlights a significant difference in the proportion by gender and age group between the pre‐COVID‐19 (January 2015–February 2020) and early post‐COVID‐19 period (June 2023–December 2024). Overall, the proportion of women and young women was higher in the early post‐pandemic period. In contrast, a comparison between the pandemic period (March 2020–May 2023) and the early post‐pandemic period (June 2023–December 2024) showed that the proportions were broadly identical, except for women and young women for overall ED and atypical ED, where proportions are significantly higher in the early post‐pandemic period. However, the proportion of young women was higher (but not significant) in the early post‐COVID‐19 period than in the pandemic period for all ED combined, except for BN, for which the proportion of men and young men was higher in the post‐pandemic period.

**Table 1:** Characteristics of patients with eating disorders.

Eating disorders (overall and by category)	Pre COVID‐19	During COVID‐19	After COVID‐19	*p*‐Value	*p*‐Value
	January 2015–February 2020	March 2020–May 2023	June 2023–December 2024		
	*N* (%)	*N* (%)	*N* (%)	Pre/after	During/after
Eating Disorders	75,585	56,069	30,967	—	—
Gender and age					
Female	60,359 (79.86)	45,766 (81.62)	25,574 (82.58)	**<0.01**	**<0.01**
0–14	5419 (7.17)	5416 (9.66)	3313 (10.7)	**<0.01**	**<0.01**
15–19	10,383 (13.74)	10,690 (19.07)	6307 (20.37)	**<0.01**	**<0.01**
20–24	6083 (8.05)	4863 (8.67)	2895 (9.35)	**<0.01**	**<0.01**
25–44	17,503 (23.16)	11,830 (21.1)	6200 (20.02)	**<0.01**	**<0.01**
45–64	12,363 (16.36)	7991 (14.25)	4213 (13.6)	**<0.01**	**<0.01**
65–99	8608 (11.39)	4976 (8.87)	2646 (8.54)	**<0.01**	0.1
Male	15,226 (20.14)	10,303 (18.38)	5393 (17.42)	**<0.01**	**<0.01**
0–14	1663 (2.2)	1092 (1.95)	613 (1.98)	**0.02**	0.74
15–19	1089 (1.44)	953 (1.7)	515 (1.66)	**<0.01**	0.69
20–24	677 (0.9)	520 (0.93)	325 (1.05)	**0.02**	0.08
25–44	3116 (4.12)	2197 (3.92)	1180 (3.81)	**0.02**	0.43
45–64	4755 (6.29)	3182 (5.68)	1532 (4.95)	**<0.01**	**<0.01**
65–99	3926 (5.19)	2359 (4.21)	1228 (3.97)	**<0.01**	0.09

Anorexia Nervosa	32,800	24,946	13,373	—	—
Gender and age					
Female	29,942 (91.29)	23,131 (92.72)	12,417 (92.85)	**<0.01**	0.65
0–14	3513 (10.71)	3484 (13.97)	1894 (14.16)	**<0.01**	0.6
15–19	7701 (23.48)	7849 (31.46)	4241 (31.71)	**<0.01**	0.62
20–24	3696 (11.27)	2909 (11.66)	1699 (12.7)	**<0.01**	**<0.01**
25–44	8213 (25.04)	5004 (20.06)	2521 (18.85)	**<0.01**	**<0.01**
45–64	4428 (13.5)	2549 (10.22)	1304 (9.75)	**<0.01**	0.15
65–99	2391 (7.29)	1336 (5.36)	758 (5.67)	**<0.01**	0.2
Male	2858 (8.71)	1815 (7.28)	956 (7.15)	**<0.01**	0.65
0–14	440 (1.34)	311 (1.25)	143 (1.07)	**0.02**	0.13
15–19	551 (1.68)	468 (1.88)	242 (1.81)	0.33	0.65
20–24	254 (0.77)	155 (0.62)	96 (0.72)	0.53	0.26
25–44	596 (1.82)	376 (1.51)	198 (1.48)	**0.01**	0.84
45–64	441 (1.34)	220 (0.88)	124 (0.93)	**<0.01**	0.65
65–99	576 (1.76)	285 (1.14)	153 (1.14)	**<0.01**	0.99

Bulimia Nervosa	9482	6147	3688	—	—
Gender and age					
Female	8380 (88.38)	5590 (90.94)	3313 (89.83)	**0.02**	0.07
0–14	292 (3.08)	322 (5.24)	225 (6.1)	**<0.01**	0.07
15–19	1504 (15.86)	1314 (21.38)	799 (21.66)	**<0.01**	0.74
20–24	1318 (13.9)	991 (16.12)	547 (14.83)	0.17	0.09
25–44	3462 (36.51)	2059 (33.5)	1178 (31.94)	**<0.01**	0.11
45–64	1477 (15.58)	740 (12.04)	460 (12.47)	**<0.01**	0.52
65–99	327 (3.45)	164 (2.67)	104 (2.82)	0.07	0.65
Male	1102 (11.62)	557 (9.06)	375 (10.17)	**0.02**	0.07
0–14	68 (0.72)	24 (0.39)	28 (0.76)	0.8	**0.02**
15–19	91 (0.96)	62 (1.01)	58 (1.57)	**<0.01**	**0.02**
20–24	89 (0.94)	54 (0.88)	36 (0.98)	0.84	0.62
25–44	396 (4.18)	197 (3.2)	135 (3.66)	0.18	0.23
45–64	319 (3.36)	158 (2.57)	84 (2.28)	**<0.01**	0.36
65–99	139 (1.47)	62 (1.01)	34 (0.92)	**0.02**	0.67

Atypical Eating Disorders	33,592	25,695	14,165	—	—
Gender and age					
Female	23,314 (69.4)	18,445 (71.78)	10,546 (74.45)	**<0.01**	**<0.01**
0–14	1783 (5.31)	1802 (7.01)	1320 (9.32)	**<0.01**	**<0.01**
15–19	2195 (6.53)	2482 (9.66)	1834 (12.95)	**<0.01**	**<0.01**
20–24	1658 (4.94)	1485 (5.78)	907 (6.4)	**<0.01**	**0.01**
25–44	6281 (18.7)	5054 (19.67)	2591 (18.29)	0.3	**<0.01**
45–64	5698 (16.96)	4228 (16.45)	2174 (15.35)	**<0.01**	**<0.01**
65–99	5699 (16.97)	3394 (13.21)	1720 (12.14)	**<0.01**	**<0.01**
Male	10,278 (30.6)	7250 (28.22)	3619 (25.55)	**<0.01**	**<0.01**
0–14	1096 (3.26)	671 (2.61)	391 (2.76)	**<0.01**	0.38
15–19	432 (1.29)	430 (1.67)	209 (1.48)	0.10	0.13
20–24	333 (0.99)	288 (1.12)	156 (1.1)	0.28	0.86
25–44	1841 (5.48)	1427 (5.55)	706 (4.98)	**0.03**	**0.02**
45–64	3528 (10.5)	2491 (9.69)	1172 (8.27)	**<0.01**	**<0.01**
65–99	3048 (9.07)	1943 (7.56)	985 (6.95)	**<0.01**	**0.03**

Binge Eating Disorders	5700	4803	3107	—	—
Gender and age					
Female	4218 (74)	3724 (77.53)	2407 (77.47)	**<0.01**	0.95
0–14	154 (2.7)	216 (4.5)	189 (6.08)	**<0.01**	**<0.01**
15–19	323 (5.67)	447 (9.31)	360 (11.59)	**<0.01**	**<0.01**
20–24	312 (5.47)	407 (8.47)	285 (9.17)	**<0.01**	0.28
25–44	1651 (28.96)	1495 (31.13)	874 (28.13)	0.41	**<0.01**
45–64	1455 (25.53)	992 (20.65)	584 (18.8)	**<0.01**	**0.04**
65–99	323 (5.67)	167 (3.48)	115 (3.7)	**<0.01**	0.6
Male	1482(26)	1079 (22.47)	700 (22.53)	**<0.01**	0.95
0–14	105 (1.84)	112 (2.33)	78 (2.51)	**0.04**	0.61
15–19	104 (1.82)	86 (1.79)	58 (1.87)	0.89	0.80
20–24	70 (1.23)	69 (1.44)	65 (2.09)	**<0.01**	**0.03**
25–44	451 (7.91)	338 (7.04)	229 (7.37)	0.36	0.57
45–64	568 (9.96)	390 (8.12)	205 (6.6)	**<0.01**	**0.01**
65–99	184 (3.23)	84 (1.75)	65 (2.09)	**<0.01**	0.27

*Note:* Values in bold indicate statistical signficance.

Among the 162,621 patients hospitalized for ED during the study period, 80.99% were female. People under the age of 25 years accounted for 38.63% of patients with ED.

As shown in Figure 1, where the difference between expected and observed trends can be seen, there was an increase in the number of young patients for ED during the pandemic period. For female patients, an increase was observed in all age groups (0–14, 15−19, and 20–24). Among men, only the 15–19 age group appeared to be affected by the pandemic.

Figure 1Evolution of the number of patients for eating disorders in the different age groups: (a) in women and (b) in men. The dotted line on the graphs is a trend line calculated between 2015 and 2019 to compare the pandemic trend expected with the pandemic trend observed.

**Figure:**
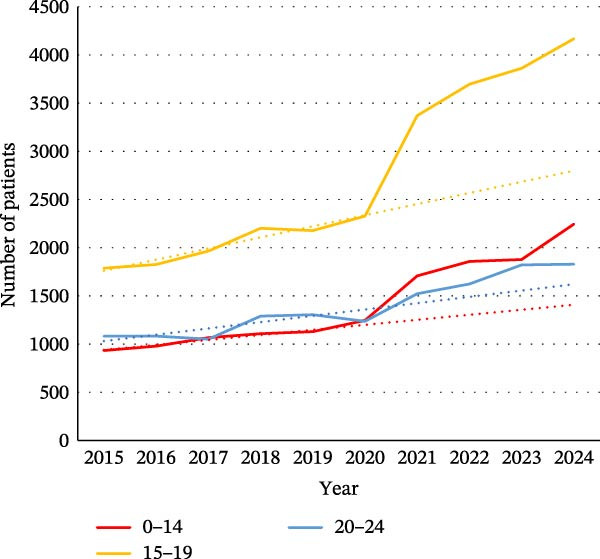
(a)

**Figure:**
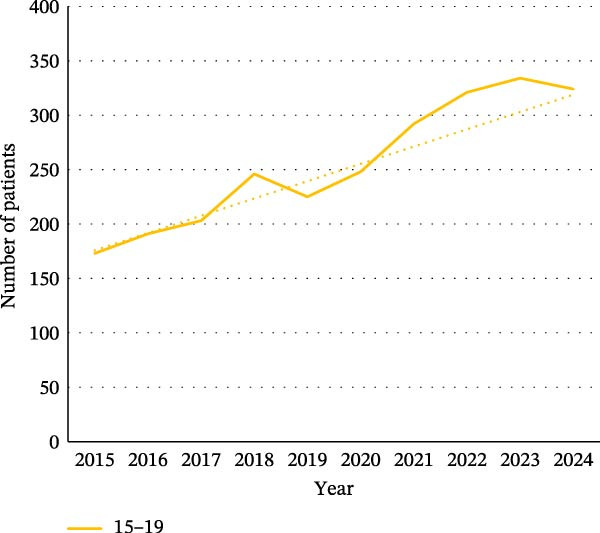
(b)

We found that each of the categories of ED was affected by the pandemic, particularly in the younger population. For female patients, the increase was observed in all age groups (0–14, 15−19, and 20–24) for each category (Figure 2). For BN, there were more hospitalized patients over the 2020–2024 period among women and men aged between 45 and 64 (Figure S2).

Figure 2Evolution of the number of patients for the different categories of eating disorders in the different age groups: (a) in women with a diagnosis of anorexia nervosa, (b) in women with a diagnosis of bulimia nervosa, (c) in women with a diagnosis of atypical eating disorders, and (d) in women with a diagnosis of binge eating disorders. The dotted line on the graphs is a trend line calculated between 2015 and 2019 to compare the pandemic trend expected with the pandemic trend observed.

**Figure:**
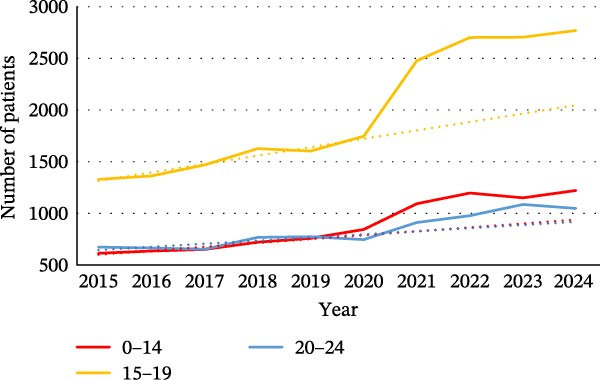
(a)

**Figure:**
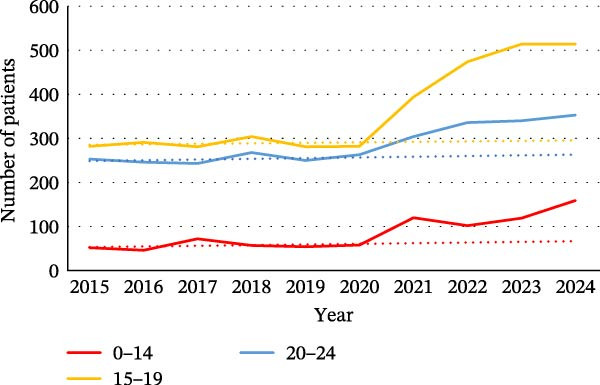
(b)

**Figure:**
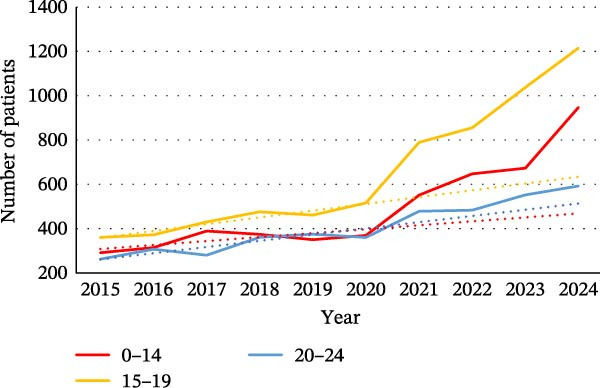
(c)

**Figure:**
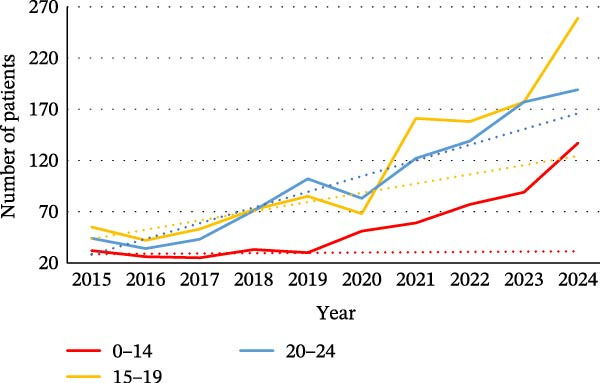
(d)

**Figure 3:**
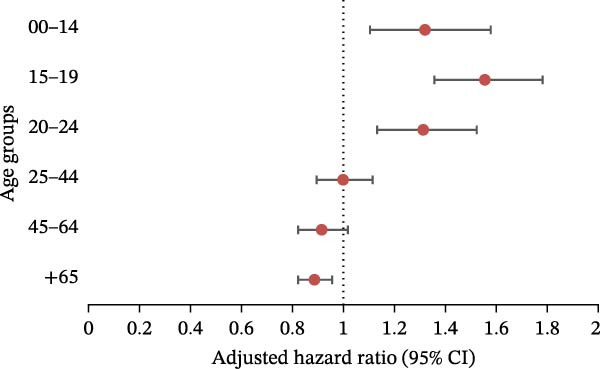
Negative binomial regression (with adjustment on sex, depression, psychiatric disorders, and Charlson score): results for eating disorders, aRR, and 95% CI in the different age groups.

A negative binomial regression model was performed for each age group to determine how the number of patients hospitalized for ED varied during the pandemic (period 2020–2024), with adjustment for sex, depression, psychiatric disorders, and Charlson score (Figure 3). The pandemic had an influence on the under‐25 population, with significant variations seen in the 0–14, 15−19, and 20–24 age groups. The 15–19 age group was the most affected, with an aRR of 1.55 (95%CI: 1.36, 1.78). The significant aRR for the 0–14 and 20−24 age groups were 1.32 (95%CI: 1.10, 1.58) and 1.31 (95%CI: 1.13, 1.52), respectively.

We performed analyses using the same models for each ED category (Figure 4). The pandemic had a significant impact on: AN (Figure 4a) in the 0–14 age group (aRR = 1.37 [1.21, 1.55]), 15–19 age group (aRR = 1.47 [1.26, 1.71]) and 20–24 age group (aRR = 1.23 [1.03, 1.45]); BN (Figure 4b) in the 0–14 age group (aRR = 1.65 [1.35, 2.02]), 15–19 age group (aRR = 1.39 [1.20, 1.60]), and 20–24 age group (aRR = 1.19 [1.02, 1.39]); atypical ED (Figure 4c) in people aged between 0 and 44 (0–14: aRR = 1.35 [1.06, 1.72]; 15–19: aRR = 1.74 [1.47, 2.05]; 20–24: aRR = 1.44 [1.22, 1.69]; 25–44: aRR = 1.13 [1.01, 1.27]); BED (Figure 4d) in people aged between 0 and 44 (0–14: aRR = 2.30 [1.89, 2.80]; 15–19: aRR = 2.11 [1.71, 2.60]; 20–24: aRR = 1.95 [1.54, 2.48]; 25–44: aRR = 1.22 [1.03, 1.46]).

Figure 4Negative binomial regression (with adjustment on sex, depression, psychiatric disorders, and Charlson score): results for each category of eating disorder, aRR, and 95% CI in the different age groups. (a) Anorexia nervosa. (b) Bulimia nervosa. (c) Atypical eating disorders. (d) Binge eating disorders.

**Figure:**
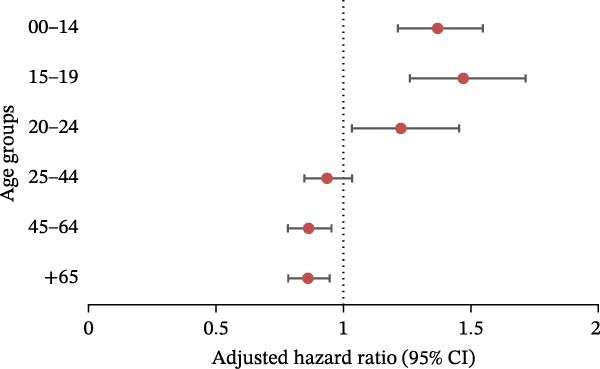
(a)

**Figure:**
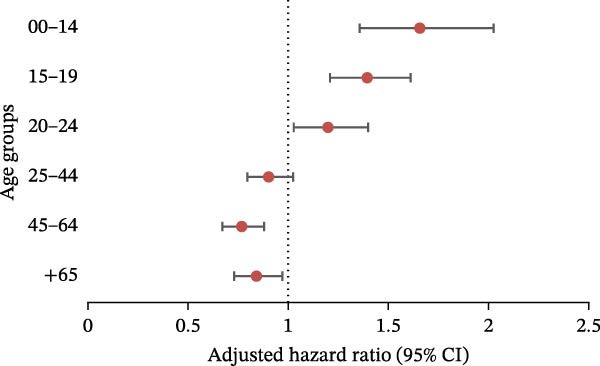
(b)

**Figure:**
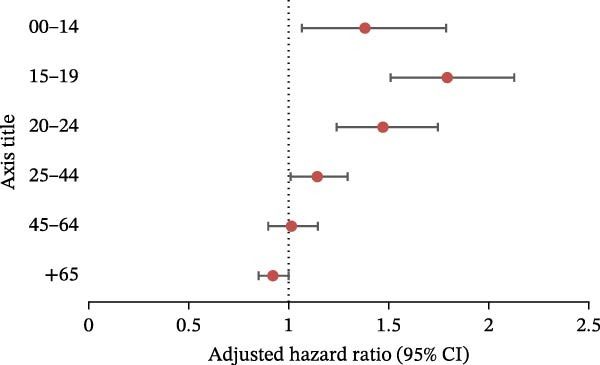
(c)

**Figure:**
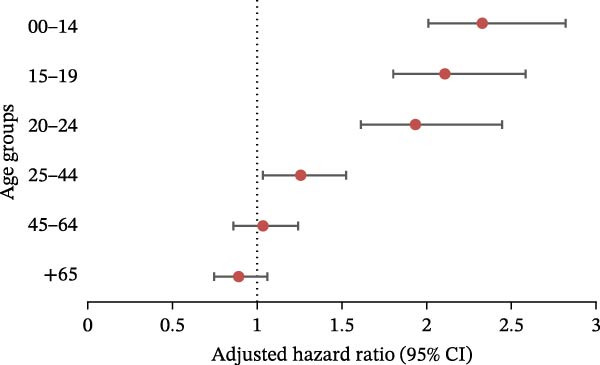
(d)

We also adjusted the models for social deprivation index, in addition to gender, depression, psychological disorders, and Charlson score. The impact of the pandemic was similar to that of the previous model (data not shown).

### 3.2. Severity of EDs

EDs appear to have worsened since the COVID‐19 pandemic in terms of medical complications (Table 2), compared to the pre‐COVID period (23.82%), with a higher rate during the COVID‐19 period (25.33%, *p*  < 0.01) but also after the pandemic (24.90%, *p*  < 0.01)).

**Table 2:** Severity of ED during the first ED stay of the year.

Severity of ED	Pre COVID‐19	During COVID‐19	After COVID‐19	*p*‐Value	*p*‐Value
	January 2015–February 2020	March 2020–May 2023	June 2023–December 2024		
	*N* (%)	*N* (%)	*N* (%)	Pre/after	During/after
Admission to intensive care	140 (0.19)	94 (0.17)	67 (0.22)	0.29	0.11
Medical complications ^∗^	18,002 (23.82)	14,204 (25.33)	7711 (24.90)	**<0.01**	0.16
Length of stay					
Mean (std)	8.38 (16.54)	8.15 (18.26)	7.55 (18.42)	**<0.01**	**<0.01**
Median [Q1‐Q3]	3 [0–9]	2 [0–8]	2 [0–7]	—	—

*Note:* Values in bold indicate statistical signficance.

^∗^Malnutrition, hypokalemia, liver failure, heart failure, hypoglycemia, kidney failure, leukopenia, neutropenia, hypothermia, change in blood pressure, and placement of a gastric tube.

Regarding suicidal ideation and/or self‐harm, among patients with an ED, 8.67% had a hospital stay involving a code of suicidal ideation and/or self‐harm within 2 years following an ED‐related admission in the pre‐COVID‐19 period, compared with 13.31% during the COVID‐19 and early post‐COVID periods (*p*‐value <0.01) (Table 3). The results were similar for AN (11.07% vs. 16.47%, *p*‐value <0.01), BN (14.8% vs. 22.57%, *p*‐value <0.01), atypical ED (6.76% vs. 11.33%, *p*‐value <0.01), and BED (5.33% vs. 8.96%, *p*‐value <0.01).

**Table 3:** Suicidal rate (suicidal ideation and/or self‐harm) after eating disorders (2‐year follow‐up).

Eating disorders (overall and by category)	a. Before COVID, inclusion year for 2‐year follow‐up: 2015–2017		b. Beginning of COVID, inclusion year for 2‐year follow‐up: March 2020–February 2021		c. COVID and early post‐COVID periods, inclusion year for 2‐year follow‐up: March 2020–December 2022			
	*N*	%	*N*	%	*N*	%	*p*‐value (a) vs (b)	*p*‐value (a) vs (c)
Eating disorders, overall	3514	8.67	1667	11.12	6407	13.31	**<0.0001**	**<0.0001**
Anorexia nervosa	2036	11.07	951	14.33	3514	16.47	**<0.0001**	**<0.0001**
Bulimia nervosa	816	14.8	299	19.67	1179	22.57	**<0.0001**	**<0.0001**
Atypical eating disorders	1172	6.76	614	8.85	2514	11.33	**<0.0001**	**<0.0001**
Binge eating disorders	132	5.33	85	7.2	366	8.96	**0.0251**	**<0.0001**

*Note:* Values in bold indicate statistical signficance.

When self‐harm and suicidal ideation are separated, the difference in proportion is significant for all EDs (overall or classified), and the proportion is higher during the COVID‐19 and early post‐COVID periods (Tables S2 and S3).

## 4. Discussion

This study is the first to explore a potential relationship between the COVID‐19 pandemic and hospitalizations for ED, offering a comparative and qualitative hospital survey based on the longitudinal analysis of 162,621 patients hospitalized for EDs in France between 2015 and 2024. The retrospective longitudinal follow‐up makes it possible to examine the trends in ED‐related hospitalizations over a 10‐year period using data from the French National Health Data System. Findings from the PMSI database revealed a particularly sharp increase during the COVID‐19 period (2020–2024) compared to the pre‐pandemic period (2015–2020), particularly among adolescents and young adults. Moreover, we saw an increase in ED associated with suicidal behavior: the rate of patients hospitalized for suicidal ideation or self‐harm within 2 years following an ED‐related admission was higher when the ED admission occurred at the beginning of the pandemic and remained elevated during the late‐pandemic and early post‐COVID periods, compared with the pre‐pandemic period. This 10‐year observational study thus provides an epidemiological overview of the evolution of EDs and severe cases with suicidal ideation and self‐harm.

The substantial rise in ED‐related hospitalizations in the COVID‐19 period, particularly among young women, aligns with global findings indicating a surge in mental health disorders during this time. A systematic review reported a 48% increase in hospital admissions across various studies, with pediatric admissions rising by an average of 83% and adult admissions by 16% compared to pre‐pandemic levels [21]. This trend has been corroborated by studies from Canada and Australia documenting significant increases in hospitalizations for AN and other EDs [22–24]. For instance, a study in Ontario found that acute ED presentations among adolescents and adults increased during the pandemic, reflecting a broader strain on mental health services [25]. Recent international studies have confirmed that the rise in the incidence of EDs observed during the COVID‐19 pandemic has persisted beyond the acute phase, with post‐pandemic prevalence rates remaining higher than expected across several countries, particularly among adolescents and young adults [26–28]. Our study confirms this general trend of worsening mental health among adolescents and young adults in the aftermath of the COVID‐19 pandemic, with a near doubling of hospital stays for EDs. Several pandemic‐related stressors, such as social isolation, disrupted daily routines, increased screen time, and heightened anxiety, likely contributed to the exacerbation of ED symptoms and the emergence of new cases [10]. School closures and limited access to extracurricular activities reduced protective social interactions, increasing vulnerability to ED development or the worsening of pre‐existing EDs [29]. The pandemic introduced unprecedented levels of stress, including fear of illness, uncertainty about the future, and economic instability [30], all of which may have triggered or aggravated ED symptoms in at‐risk individuals.

Our findings are consistent with prior inpatient and clinical cohort studies reporting pandemic‐related changes in ED severity, clinical presentation, and service delivery. Several hospital‐based cohorts have shown that patients admitted during the COVID‐19 period, particularly adolescents with AN, presented with greater clinical severity at admission, including lower BMI, increased medical complications, and higher psychiatric comorbidity compared with pre‐pandemic cohorts [31, 32]. In parallel, substantial disruptions in outpatient care and delayed referrals have been reported during the pandemic and may have contributed to a higher proportion of acute and medically severe inpatient presentations [33, 34]. Within this clinical context, our nationwide findings extend previous inpatient observations by demonstrating, at the population level, a sustained increase in both severe ED presentations and subsequent hospitalizations for self‐harm and suicidal ideation, suggesting that the pandemic not only increased the volume of admissions but also altered the clinical profile of hospitalized patients [8].

Beyond the population‐level increase in hospital admissions, several studies suggest a qualitative shift in the clinical profiles of patients hospitalized for EDs during the COVID‐19 pandemic. The notion of increased clinical severity at admission was specifically highlighted in the adolescent population by Schreyer et al. [32], who observed a parallel rise in depressive symptoms and hospital admissions. A review by Devoe et al. [21] also showed, in individuals aged 13–42 years, a qualitative increase in anxiety and depressive symptoms in addition to a quantitative rise in ED‐related hospitalizations. Consistent with these clinical observations, our findings further indicate that patients admitted for EDs in the early post‐COVID period present with greater clinical and biological severity than those admitted before the pandemic. In this context, the present study, which reports increases in hospitalizations for self‐harm and suicidal behaviors as well as a rise in medical complications among individuals with EDs, suggests a worsening of clinical and biological profiles in the post‐pandemic period compared with the pre‐pandemic era. Although caution is warranted when interpreting these findings, the clinical and epidemiological aggravation of EDs associated with the COVID‐19 pandemic appears to be a hypothesis supported by numerous studies.

In particular, the pandemic has presumably reinforced the importance placed on physical appearance, exercise, and weight control, which have been described as a “psychological stress induced by the pandemic”[7]. Increased screen time during lockdowns exposed individuals, particularly adolescents and young adults [35], to idealized body images and diet culture content on social media, exacerbating body dissatisfaction and disordered eating behaviors. Several studies have highlighted the link between social media use and deteriorating mental health, as well as its role in negative body image perceptions among young women [36, 37]. Other studies have suggested that the pandemic was associated with a deterioration in body image, along with food insecurity and stockpiling behaviors, which may have also contributed to binge eating and restrictive eating patterns [38]. Our findings appear to illustrate this worsening trend in the COVID‐19 period, particularly in BED, with a dramatic increase in hospital prevalence, nearly quadrupling compared to pre‐pandemic levels. Lockdown periods likely intensified family tensions, adding stress and reducing adolescent autonomy, both known risk factors for EDs. Moreover, the disruption of outpatient mental health services and school‐based support programs during the pandemic likely delayed early detection and intervention, resulting in more severe cases requiring hospitalization.

We already previously observed that, while the strict March–May 2020 lockdown was associated with a significant reduction in self‐harm rates in the whole population, a significant rise in self‐harm hospitalizations in adolescent females started in December 2020–January 2021 [6]. In France, the first wave of COVID‐19 began on February 23, 2020, leading to an initial 2‐month lockdown from March 17 to May 11, 2020, with the epidemic peaking in mid‐April 2020. During this period, the population was forced to stay at home. Although necessary to reduce the spread of COVID‐19, this lockdown had a considerable impact on the lifestyle and daily habits of most of the population [39]. A second, less strict lockdown lasting 1 month was implemented from October 28 to November 28, 2020. It was renewed between April 3 and May 3, 2021, with different conditions (short daytime outings allowed, in‐person work maintained if teleworking was impossible), resulting in changes to professional activities and leisure activities. Our current results highlight the apparently increased association between EDs and suicidal behavior (self‐harm and suicidal ideation) in the COVID‐19 period (March 2020– December 2022) compared to the pre‐pandemic period (2015–2017). This underscores the severe psychiatric burden of EDs, which extends beyond eating‐related concerns to encompass complex psychiatric comorbidities. Several hypotheses underlie this association, primarily the high prevalence of depressive comorbidities in EDs. For instance, studies have shown that adolescents hospitalized during the pandemic reported higher levels of EDs and depressive symptoms compared to those admitted before the pandemic [32]. Low self‐esteem and negative body image, both strongly associated with EDs, have also been linked to suicidal ideation and behaviors. The major psychosocial impact of COVID‐19 may have exacerbated these phenomena, increasing the suicidal risk—a finding reinforced by our study. Additionally, environmental stressors may have interacted with biological and genetic vulnerabilities, further compounding suicide risk in individuals with EDs [14]. As discussed above, a systematic review suggested that while ED‐specific psychopathology did not change during the pandemic, anxiety and depression symptoms increased significantly [40]. These results have also been replicated in a similar study [41]. The rise in suicidal behaviors (both self‐harm and suicidal ideation) observed in our study in individuals with EDs appears to confirm this hypothesis, pointing to an impaired ability to adapt to adversity caused by the health crisis and underlying pathophysiological mechanisms in EDs, that is, a high vulnerability to stress. These findings underscore the critical need for integrated mental health assessments in ED management, including routine screening for suicidal thoughts and behaviors [42]. Beyond documenting the rise in hospitalizations for ED during and after the COVID‐19 pandemic, these findings echo broader international reflections on preparedness for future health crises. Several authors have emphasized that the pandemic acted as a “stress test” for mental health systems, revealing the fragility of early detection pathways, the need for flexible care delivery models (including telepsychiatry), and the importance of maintaining continuity of specialized care during periods of social restriction through personalized medicine [40]. Strengthening epidemiological surveillance, improving coordination between primary care and specialized services [43], and investing in prevention programs targeting vulnerable populations, particularly adolescents and young adults, should therefore be considered key priorities to mitigate the long‐term consequences of future pandemics on mental health and EDs.

A noteworthy aspect of this study is the 10‐year observation period, allowing for the identification of long‐term hospitalization trends. Our findings reveal both an immediate surge in hospitalizations at the beginning of the COVID‐19 period (2020–2021) and a sustained increase beyond the pandemic, persisting until the end of 2024 for most EDs (except for BN and atypical ED). However, caution is warranted when interpreting the underlying causes of this sustained rise. Indeed, the timing of available analyses, with post‐COVID observations still relatively limited, does not yet allow for definitive conclusions regarding an irreversible and ongoing impact of the pandemic on the emergence of EDs. For reasons discussed earlier, including the rise of social media, disruption to daily activities or social isolation [44], the increase in EDs among young people appears to be a societal trend that predated the COVID crisis, likely amplified by the pandemic and its associated restrictions. However, very recent studies suggest that ED prevalence remains particularly high in certain population groups in the post‐COVID period, albeit with a slight overall slowdown [45]. In addition, the pathophysiology of EDs remains highly complex and multifactorial, with varying prevalence across different countries and cultures [46], making it difficult to draw definitive conclusions. Moreover, the continued increase in post‐lockdown hospitalizations could also reflect a destigmatization trend. Indeed, a greater willingness to seek help, facilitated by improved verbalization of mental health difficulties, may have contributed to the rise in hospital admissions. Some authors have hypothesized that hospitalization rates are associated with help‐seeking behaviors, particularly among children and young individuals [47]. The mental health difficulties exacerbated by the pandemic may also have provided an opportunity for children and parents to discuss more openly, leading to more substantial care [48]. The observed stable upward trend in ED‐related hospitalizations underscores the fact that EDs should not be considered a niche pathology but rather a significant public health issue [49]. In this sense, the present study provides evidence that should encourage greater attention to the mental health of young women, while also acknowledging the needs of young men. Another key strength of this study lies in its broad epidemiological approach. Our data confirm a significant gender disparity, with females exhibiting higher hospitalization rates across all age groups [50], particularly for AN. Interestingly, while ED prevalence among males remains lower, the observed upward trend in male hospitalizations suggests that EDs may frequently go undetected in men, and they therefore warrant increased clinical attention.

We recognize that our study has some limitations. Firstly, clinical symptoms and specific patient characteristics are not necessarily identifiable via ICD‐10 codes in the PMSI database, which may limit the interpretation of results and potential mechanisms. Additionally, many patients who self‐harm are not hospitalized. Our study only considered hospital admissions, which may have underestimated the potential association, in particular between hospitalizations for ED and subsequent self‐harm. For instance, a French national survey found that around 40% of individuals do not go to the hospital following a self‐harm episode [51]. Moreover, as shown in our validation study [52], even if documented self‐harms usually reflect true self‐harming episodes, hospital administrative databases may underestimate self‐harm occurrence. However, we believe that this underestimation should not have hindered the comparison of the pre‐COVID‐19 period with the period at the start of COVID‐19 (during which we found an increase in suicidal disorders among individuals with EDs), as this underestimation has probably remained similar or even increased due to the reduction in hospital admissions at the start of the COVID‐19 period. The ICD‐10 coding used for suicidal ideation may not be reliable in the PMSI. To our knowledge, no validation study of this coding has been carried out. Nevertheless, in France, the coding (R45.8) is used by the French Public Health Agency in its various local and national reports (https://www.santepubliquefrance.fr/content/download/760320/document_file/bullnat_conduites_suicidaires_20251010.pdf https://www.santepubliquefrance.fr/content/download/506756/3770345). Furthermore, to compensate for the lack of reliability of this coding, we conducted the same analyses without this coding, which yielded similar results. Finally, in the medico‐administrative databases, it is not possible to identify the level of social restrictions and economic difficulties. Nevertheless, this retrospective analysis of over 329,501 hospital stays for ED between 2015 and 2024 provides valuable insights into the continued rise in hospital admissions for EDs and associated suicidal behaviors (self‐harm and suicidal ideation) in the COVID‐19 era.

## 5. Conclusion

Our findings underscore the urgent need for targeted prevention strategies and early intervention programs, particularly for adolescents and young adults. The surge in ED hospitalizations during the COVID‐19 pandemic reflects a broader mental health crisis that was exacerbated during this period. Addressing this issue requires a multifaceted approach that combines public health policies, clinical interventions, and ongoing research to better understand and mitigate the long‐term consequences of the COVID‐19 pandemic on mental health. Public health initiatives should focus on increasing awareness of ED and depressive symptoms, reducing stigma, and promoting mental health literacy.

## Author Contributions

Conceptualization: J. C. Chauvet‐Gélinier, J. M. Pinoit, and C. Quantin. Methodology: E. Lajeune, J. Cottenet, and C. Quantin. Validation: C. Quantin. Formal analysis: J. Cottenet and E. Lajeune. Writing – original draft preparation: J. C. Chauvet‐Gélinier and C. Quantin. Writing – review and editing: all authors. Supervision: C. Quantin.

## Funding

The authors received no specific funding for this work.

## Disclosure

All authors have read and agreed to the published version of the manuscript.

## Ethics Statement

This study was conducted according to the guidelines of the Declaration of Helsinki and approved by the National Committee for Data Protection: Declaration of Conformity to the methodology of Reference 05 obtained on 7 August 2018 under the Number 2204633 v0.

## Conflicts of Interest

The authors declare no conflicts of interest.

## Supporting Information

Additional supporting information can be found online in the Supporting Information section.

## Supporting information


**Supporting Information** Table S1: International Classification of Diseases (ICD‐10) codes used. Table S2: Suicidal ideation after eating disorders (two‐year follow‐up). Table S3: Self‐harm after eating disorders (two‐year follow‐up). Figure S1: Evolution of the number of patients with an eating disorder Figure S2: Evolution of the number of patients for bulimia nervosa in the 45–64 year age groups.

## Data Availability

The use of the data from the French hospital database by our department was approved by the National Committee for Data Protection. We are not allowed to transmit these data. PMSI data are available for researchers who meet the criteria for access to these confidential French data (this access is subject to the approval of the National Committee for Data Protection) from the national agency for the management of hospitalization (ATIH—Agence technique de l’information sur l’hospitalisation; 117 boulevard Marius Vivier Merle—69329 Lyon Cedex 03).
