# Supplementary material for: Changes in Hospitalization for Eating Disorders and Related Suicidal Risk, Following COVID‐19 Pandemic
Source: Depress Anxiety. 2026 Mar 3;2026:3364565. doi: 10.1155/da/3364565 (PMC12956842; doi:10.1155/da/3364565)
Supplement: Supplementary file 1 — Supporting Information Table S1: International Classification of Diseases (ICD‐10) codes used. Table S2: Suicidal ideation after eating disorders (two‐year follow‐up). Table S3: Self‐harm after eating disorders (two‐year follow‐up). Figure S1: Evolution of the number of patients with an eating disorder Figure S2: Evolution of the number of patients for bulimia nervosa in the 45–64 year age groups. [file DA-2026-3364565-s001.docx]

Supplementary Figure 1: Evolution of the number of patients with an eating disorder

b)

a)


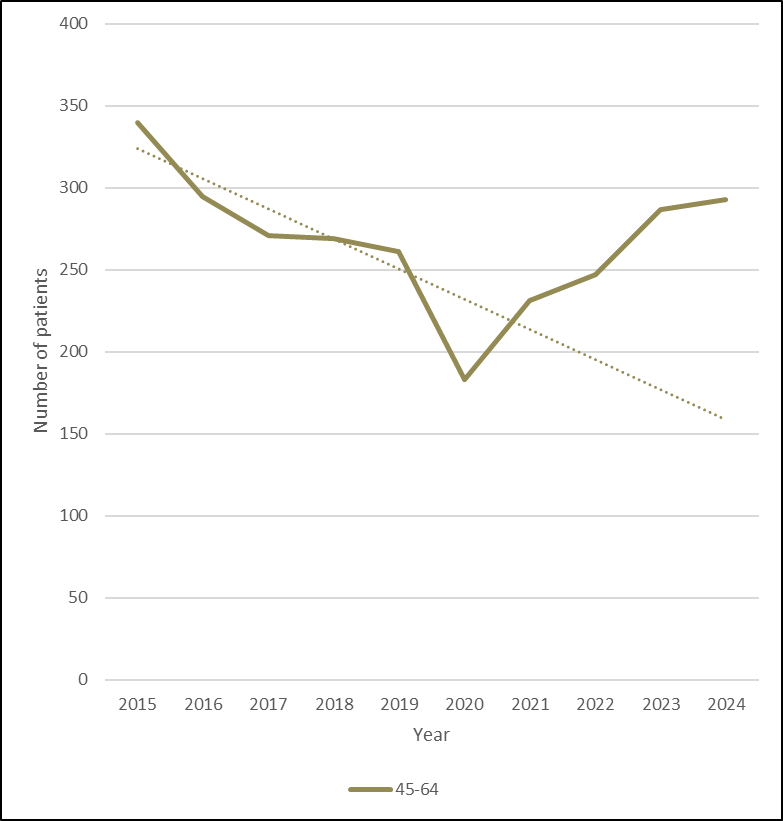

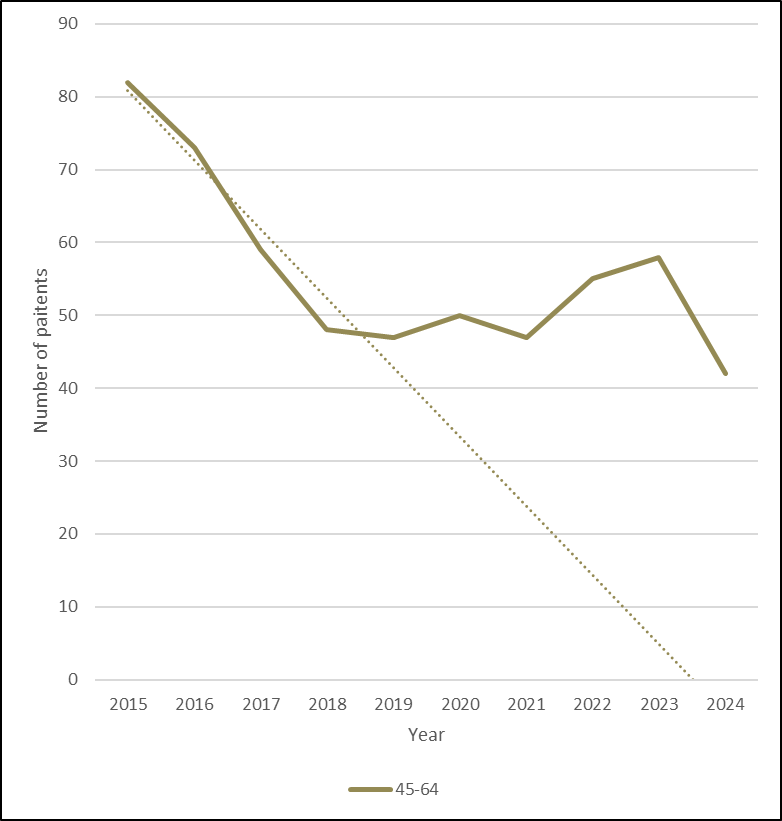


Supplementary Figure 2: Evolution of the number of patients for bulimia nervosa in the 45-64 year age groups: a) in women b) in men. The dotted line on the graphs is a trend line calculated between 2015 and 2019 to compare the pandemic trend expected with the pandemic trend observed

Supplementary Table 1: International Classification of Diseases (ICD-10) codes and Classification Commune des Actes Médicaux (CCAM) codes used.

|  | **ICD-10** |
| --- | --- |
| Eating disorders | F50 |
| Anorexia nervosa | F500 F501 |
| Bulimia nervosa | F502 F503 |
| Atypical eating disorders | F505 F508 F509 |
| Binge eating disorders | F504 |
| Self-harm | X60 – X84 |
| Suicidal ideation | R458 |
| Depression | F32 F33 |
| Psychiatrique disorders | F without F32 F33 F50 F00 F01 F02 F03 F051 |
| Malnutrition | E43 |
| Hypokalemia | E876 |
| Liver failure | R740 |
| Heart failure | R00 |
| Hypoglycemia | E15 E161 E162 |
| Kidney failure | N17 N19 |
| Leukopenia | R72 |
| Neutropenia | D70 |
| Hypothermia | R680 T68 |
| Change in blood pressure | I95 (without I952) R031 |
|  | **CCAM** |
| Gastric tube insertion | HFLE001 HFLH001 |

Supplementary Table 2: Suicidal ideation after eating disorders (two-year follow-up)

| 2 | Before COVID, inclusion year for two-year follow-up:  2015-2017 | | COVID and early post-COVID periods, inclusion year for two-year follow-up:  march 2020 - dec 2022 | |  |
| --- | --- | --- | --- | --- | --- |
|  | N | % | N | % | p-value |
| Eating disorders, overall | 885 | 2.18 | 3103 | 6.45 | **<.0001** |
| Anorexia nervosa | 447 | 2.43 | 1583 | 7.42 | **<.0001** |
| Bulimia nervosa | 247 | 4.48 | 568 | 10.87 | **<.0001** |
| Atypical eating disorders | 341 | 1.97 | 1340 | 6.04 | **<.0001** |
| Binge eating disorders | 43 | 1.74 | 199 | 4.87 | **<.0001** |

Supplementary Table 3: Self-harm after eating disorders (two-year follow-up)

| 3 | Before COVID, inclusion year for two-year follow-up:  2015-2017 | | COVID and early post-COVID periods , inclusion year for two-year follow-up:  march 2020 - dec 2022 | |  |
| --- | --- | --- | --- | --- | --- |
|  | N | % | N | % | p-value |
| Eating disorders, overall | 2933 | 7.24 | 4059 | 8.43 | **<.0001** |
| Anorexia nervosa | 1748 | 9.5 | 2282 | 10.69 | **<.0001** |
| Bulimia nervosa | 691 | 12.54 | 746 | 14.28 | **0.0079** |
| Atypical eating disorders | 944 | 5.44 | 1514 | 6.83 | **<.0001** |
| Binge eating disorders | 99 | 4 | 219 | 5.36 | **0.0127** |
